# Identification and expression pattern of aluminium-responsive genes in roots of rice genotype with reference to Al-sensitivity

**DOI:** 10.1038/s41598-023-39238-8

**Published:** 2023-07-27

**Authors:** Bijoya Bhattacharjee, Akib Ali, Narendra Tuteja, Sarvajeet Gill, Arunava Pattanayak

**Affiliations:** 1 Division of Crop Science, ICAR Research Complex for NEH Region, Barapani, Meghalaya India; 2 International Centre for Genetic Engineering and Biotechnology (ICGEB), New Delhi, India; 3 Centre for Biotechnology, Maharishi Dayanand University, Rohtak, Haryana India; 4 ICAR –IIAB, Ranchi, India

**Keywords:** Biotechnology, Molecular biology, Plant sciences

## Abstract

Aluminium (Al) is the third most abundant element in the Earth's crust. Globally, acidic soil occupies 30–40% of ice-free land areas; Al toxicity is a major threat to crops. The first symptom of Al toxicity is the inhibition of root growth followed by poor root hair development, swollen root apices, necrosis of leaves and reduced yield. Although Rice (*Oryza sativa*) is an Al toxicity tolerant crop, it shows considerable variations among rice genotypes to Al exposure. Therefore, it is pertinent to understand Al toxicity and underlying mechanisms for Al tolerance in Rice. In the present study, 63 rice genotypes screened under Al stress showed significant variations of root growth. Expression stability of endogenous control genes (ECGs) revealed sulphite reductase (*SR*) as the most stable ECG that can be used as a reference gene for quantitative real-time PCR (qRT-PCR). Expression patterns of Al-responsive genes suggest genes associated with cytoskeletal dynamics, metabolism, and ion transporter could play significant roles in Al adaptation and tolerance in rice. The results showed Motodhan, Vietnam-1, Yimyu and N-861 as Al-toxicity tolerant, while Lespah, RCPL-13, VL-31329, and UPR2919-141-1 as most Al-sensitive genotypes among the studied rice lines cultivated in North-East India.

## Introduction

Aluminum (Al) toxicity is a significant constraint for crop production on highly acidic soils. At soil pH ≤ 5, rhizotoxic Al^3+^ ions are solubilized into the soil solution dramatically inhibiting root growth & development, which ultimately leads to a significant reduction in crop yields^1^. Al^3+^ is the most toxic form among different Al ion species. Al^3+^ naturally exists in acidic soils. However, the toxic effect of Al^3+^ is influenced by the concentration of the metal. It varies depending on the species of plant, including its genotype within the same species, its physiological age, its growing environment, and the time period of Al^3+^ exposure^2^. In acidic soils, Al^3+^ also reacts with soluble phosphorus (available to plants) and converts it to insoluble aluminium phosphate (not available to plants) thus leading to P deficiency in plants. Al toxicity also affects seed germination, inhibiting shoot growth by interfering with nutrient uptake, specifically of calcium magnesium and phosphorus^3^. It is known that seed germination, seedling and reproductive stages are the most critical stage of any crop that determines the productivity of that species. Understanding the physio-molecular changes that occur within these stages enhance the knowledge of the complex mechanism of plant’s adaptability to different environments factors. Therefore, we performed our preliminary assessment of Al toxicity on the seed germination and seedling stages of different genotype in rice.

It has been reported that different species exhibit different levels of Al tolerance; eg. *Oryza sativa* is approximately two to five times more tolerant than wheat, sorghum, or maize^4^ which is mediated by Al-responsive gene expression^4^. The identification and characterization of genes, transcription factors (TFs) and regulatory proteins (e.g., protein kinases) revealed that the transcription of Al-tolerance genes is likely regulated by a complex mechanism. This mechanism involves repressors and activators, co-regulation with other Al-tolerance genes^5,6^, and crosstalk with mechanisms controlling other stress responses. Therefore, a more comprehensive characterization of these complex regulatory mechanisms may be useful for accelerating the breeding of Al-tolerant crops. Based on the evidence that Al toxicity tolerance is an inducible process and rice is an Al-toxicity tolerant crop, this assumption has been the driving force for a number of molecular investigations with various approaches for Al-responsive genes from rice that may help to further determine Al-toxicity tolerant genes. Several quantitative trait loci (QTL) such as *ART1, STAR1, STAR2, Nrat1, OsFRDL4, OsALS1, OsMGT1, ASR5* and *ART2* to the Al toxicity tolerance had been cloned from different populations of rice^7^. However, due to the high level of Al-tolerance complexity and immense genetic diversity, more studies are needed to understand the genetic mechanisms of Al-tolerance in rice.

In India, North Eastern (NE) region is considered a centre of diversity and the most probable origin of rice^8^. Rice is the most important food grain of NE India accounting for around 80% of the food grain production. It is widely cultivated in upland, lowland, Juhm and deep-water conditions^9^. However, Al toxicity resulting from excessive rainfall in the NE region is responsible for removing basic cations over a long period of time and leading to increasing acidity of the soil^10^. Accountable research has been carried out to decipher the molecular mechanisms of Al toxicity in rice, however, most of the experiments were restricted to few genotype and therefore, focused research on diverse rice genotype cultivated in this region will be an effective way to find out potent Al toxicity tolerant genotype and to understand and interpret the Al toxicity in rice.

The objective of this study was to test the hypothesis that identification of new donors from the vast diversity of rice germplasm of NE India with desirable traits for Al stress tolerance may provide a very important source for rice breeding under the acid soil conditions of this region. There is a wide genotypic variation in tolerance to Al stress in the acidic soils (moderate to strong) of this region. Diversity in germplasm/genotypes for performance of any trait is often very significant, particularly with respect to biotic and abiotic stress. Therefore, it is important to consider the diversity and origin of genotypes being compared when studying the genetics, physiology and gene expression at molecular level for Al tolerance in rice. Since the root system provides the primary effect of any metal stress, the present research aims to provide a view of the effect of Al toxicity on the root growth parameters such as RTI, RRE %, RRWC and Al-responsive gene expression profiles between potent Al—toxicity tolerant and Al- sensitive genotype to elucidate molecular mechanisms that are responsible for the sensitivity differences. Furthermore, it has been noted in the literature that different reference genes can change the outcomes of a quantitative study^11^. Accurate qRT-PCR results can only be achieved if the normalization standards used are abundant and stably expressed genes. The tested potential reference genes are compared and ranked using the most popular computational tools at the moment viz. geNorm, RefFinder and comparative -Ct technique to evaluate the expression stability of candidate reference genes and to define the optimal single reference gene for all data sets (the complete set of samples).

## Results

### Germination assay to determine optimal Al concentration to study Al sensitivity in rice

**Figure 1:**
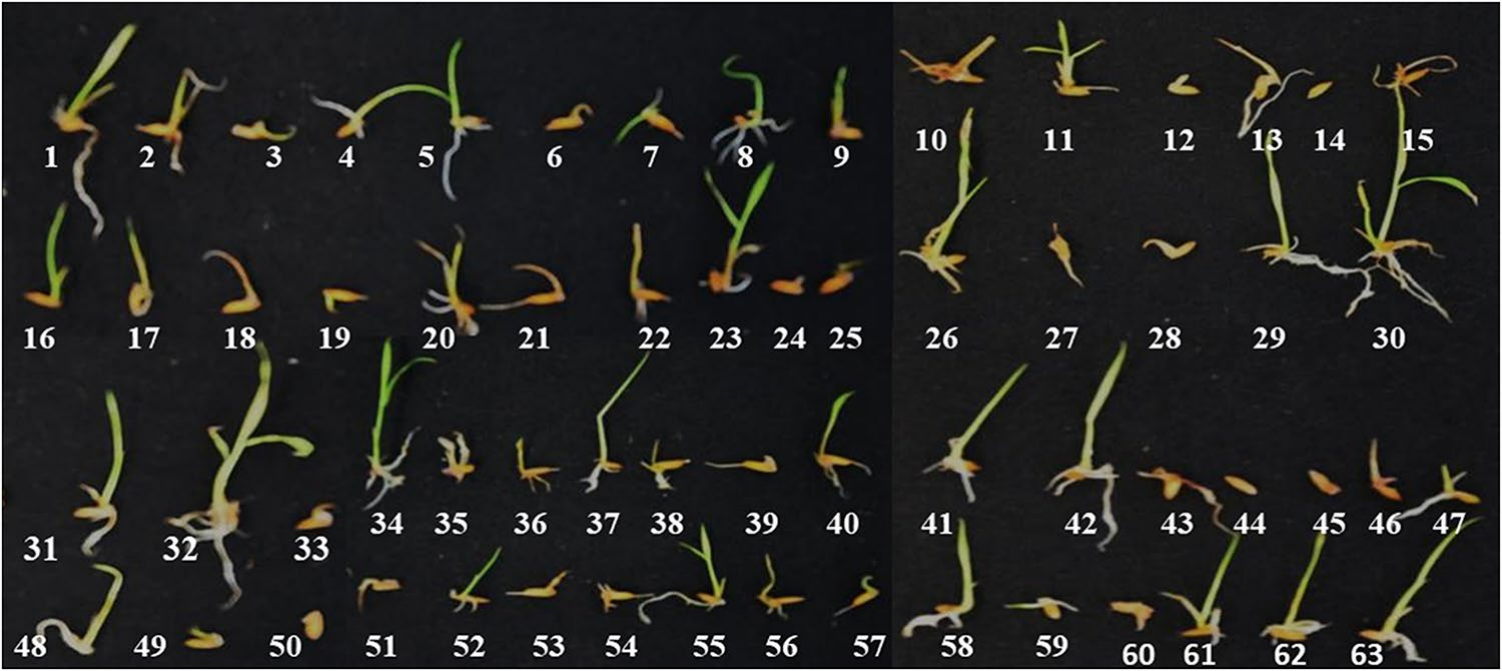
Seed germination analysis of rice genotype in nutrient solution supplemented with 200 μM Aluminum chloride; rice genotype: 1. ANJALI, 2. CHANKIMASO, 3. MOIRAMSBHI, 4. COL- 4, 5. CHING, 6. VL-31329, 7. IR1552, 8. SHAKU, 9. MICHIYING, 10. ASSAM, 11. MOTODHAN, 12. BHALUM—3, 14. VR-14, 15. LESPAH, 16. VL-31,331, 17. ZAM 18. SANRI FIIRII 19. PANCOAS 20. BHALUM – 1, 21. UPR 2992-17-3-1, 22. UPR 2919-14-1-1, 23. LIKHAMO, 24. ASUKNI MAGHOWA, 25. IORO, 26. LONGPA, TSUK, 27. MERANGKONG, 28. KONPEMO, 29. MICHIYING, 30. DHAO TIPNUAKULON, 31. RCPL- 13, 32. YIMYU, 33. MANGE, 34. IORO 35. N-861, 36. VIETNAM-1, 37. KHASHA, 38. HAHSHO, 39. TSAKNAK, 40.KHOUGJAI PHOU, 41. KASALATH, 42. TSAMU FIIRII, 43. POSIMOT, 44. VIETNAM – 3, 45. GOBINDOBHOG, 46. RCPL-1-185, 47. BANG NAYK, 48. SKAU-390 49. KALOJEERA 50. AAHA, 51. SILKY RICE, 52. KENASU KEDOWA, 53. KOYABO, 54. AKIYIUTI ASHE 55, EPYO, 56. IR 72, 57. SATABDI, 58. MEYISAO, 59. NUNG KHUM, 60. BPT—5204, 61. YIMYA MAPOK 62. SANG CHANG, 63. IDAW.

The germination test was performed for all 63 rice genotypes (Supplemental Table 1) on germination paper soaked in distilled water with or without aluminum chloride. Of the different Al concentrations tested, the 200 μM Al treatment showed significant differences. This difference in seed germination was significant enough to be detected visually (Fig. 1). It was observed that root growth in the germinating seeds of the tolerant genotypes such as Motodhan, Vietnam-1, Yimyu and N-861 was not significantly affected by the addition of 200 μM Al, while in the other genotypes root growth was significantly reduced, with a drastic reduction in the susceptible genotype (Fig. 1).

### Effect of Al (200 μM) on shoot and root growth of rice in hydroponic culture

**Figure 2:**
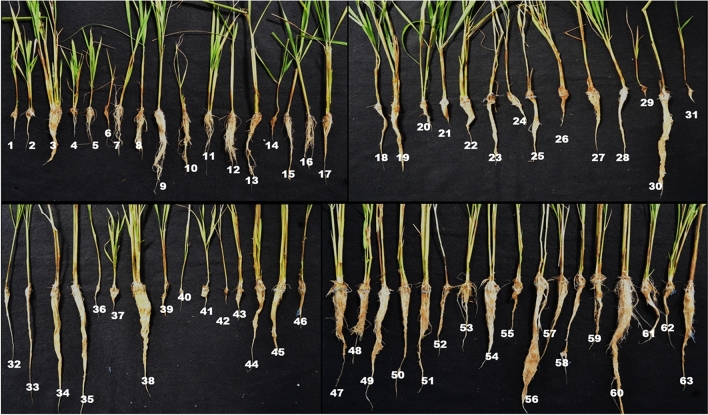
Root diversity studies of rice germplasm /varieties in hydroponics for screening of Aluminum toxicity at 200 μM Aluminum concentration ; rice genotype: 1. LESPAH, 2. RCPL- 13, 3. ANJALI, 4. MOIRAMSBHI, 5. COL- 4, 6. CHANKIMASO, 7. CHING 8. IR1552, 9. SHAKU 10. IORO, 11. SKAU-390, 12. VR-14, 13. BHALUM—3, 14. VL- 31,331, 15. ZAM, 16. UPR 2992-17-3-1, 17. PANCOAS, 18. ASUKNI MAGHOWA, 19. ASSAM, 20. LONGPA TSUK, 21. MICHIYING, 22. BANG NAYK, 23. YIMYA MAPOK, 24. SANG CHANG, 25. MEYISAO, 26. NUNG KHUM, 27. AKIYIUTI ASHE, 28. KASALATH 29. 30. BHALUM -2, 31. VL-31329, 32. KHASHA, 33. KALOJEERA, 34. KHOUGJAI PHOU, 35. POSIMOT, 36. UPR 2919-14-1-1, 37. SANRI FIIRII, 38. MOTODHAN, 39. TSAMU FIIRII, 40. BHALUM – 1, 41. MERANGKONG, 42. KONPEMO, 43. IR 72, 44. BHALUM – 4, 45. LIKHAMO, 46. DHAO TIPNUAKULON, 47. VIETNAM-1, 48. AAHA, 49. SILKY RICE, 50. KENASU KEDOWA, 51. KOYABO, 52. MANGE, 53. TSAKNAK, 54, EPYO, 55. HAHSHO, 56. YIMYU, 57. MOMCHING, 58. BPT—5204, 59. SATABDI, 60. N-861, 61. SAHABHAGI DHAN, 62. GOBINDOBHOG, 63. IDAW.

In the present study, Al-induced suppression of root growth was very pronounced in most of the treated rice lines in terms of relative root length (Fig. 2). The relative root growth of rice plants grown with 200 μM Al was not affected in a few rice lines such as Motodhan, Vietnam-1, Yimyu, and N-861. On the other hand, in Lespah, RCPL- 13, VL-31,329 and UPR 2919-141-1 lines, root length was greatly reduced by 200 μM Al treatment. Lespah showed a maximum reduction and Motodhan a maximum increase in relative root length compared with other rice lines across different time periods and Al concentrations (Supplemental Fig. 1).

The sensitivity of rice genotypes was checked and root growth was evaluated every 12 h. The presence of Al in the hydroponic medium significantly decreased the fresh weight and dry weight of roots in the sensitive cultivars at 12 and 24 h, as revealed by Bonferroni post-hoc analysis in two-way analysis ANOVA (*p* < 0.05). Due to their significant reduction in dry and fresh weight, Lespah, RCPL-13, VL-31,329, and UPR 2919-141-1 genotypes were more susceptible to Al, which was evident after 24 h. Motodhan, Vietnam-1, Yimyu, and N-861 genotypes were almost unaffected by Al toxicity in terms of fresh and dry matter accumulation in roots even when exposed to Al stress for 24 h (Supplementary Fig. 1). This again demonstrates that Al phytotoxicity leads to the largest differences in dry matter accumulation among cultivars. The decrease in root fresh weight due to the increase in Al concentration showed a similar trend to that of root dry weight.

### Effect of Al on root tolerance index (RTI)

**Figure 3:**
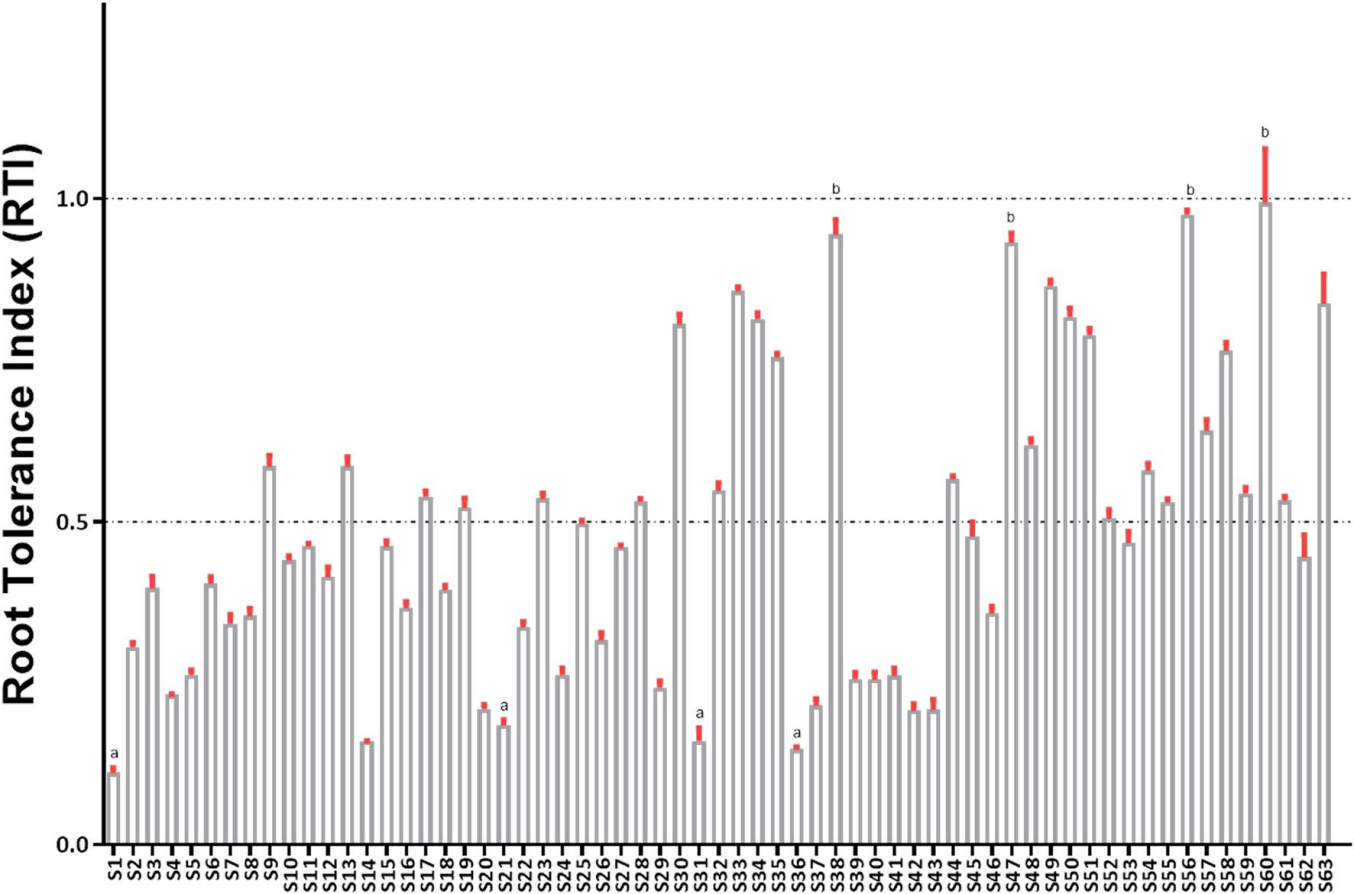
Root tolerance index (RTI) screened for 63 rice genotypes grown in presence of 200 µM Al stress calculated as the maximum root length of the treated sample divided by the maximum root length in the control. Different letters indicate significant difference at *P* < 0.05, according to the Duncan’s test. (Details of S1 to S63 is given in supplementary Table 1).

Significant differences in RTI were observed among the 63 rice lines studied. A large reduction in root length was observed at an Al concentration of 200 μM in the nutrient solution. The RTI -values at 200 μM Al concentration ranged from (0.208–0.996) among the rice lines used in this experiment. According to the RTI-values at 200 μM Al concentration, Motodhan, a highland seedling, was the most tolerant genotype (Fig. 3). The other tolerant genotypes were Vietnam-1, Yimyu, and N-861. It is noteworthy that the RTI -values of these 4 rice genotypes were greater than 0.987, indicating that the Al concentration, which is cytotoxic to the susceptible rice genotype, can stimulate root growth of the tolerant genotype. Lespah, RCPL- 13, VL-31,329 and UPR 2919-141-1 were also highly sensitive to Al. The rest of the genotypes/varieties were found to be moderately tolerant to Al toxicity.

### Effect of Al stress on relative root elongation (RRE)

**Figure 4:**
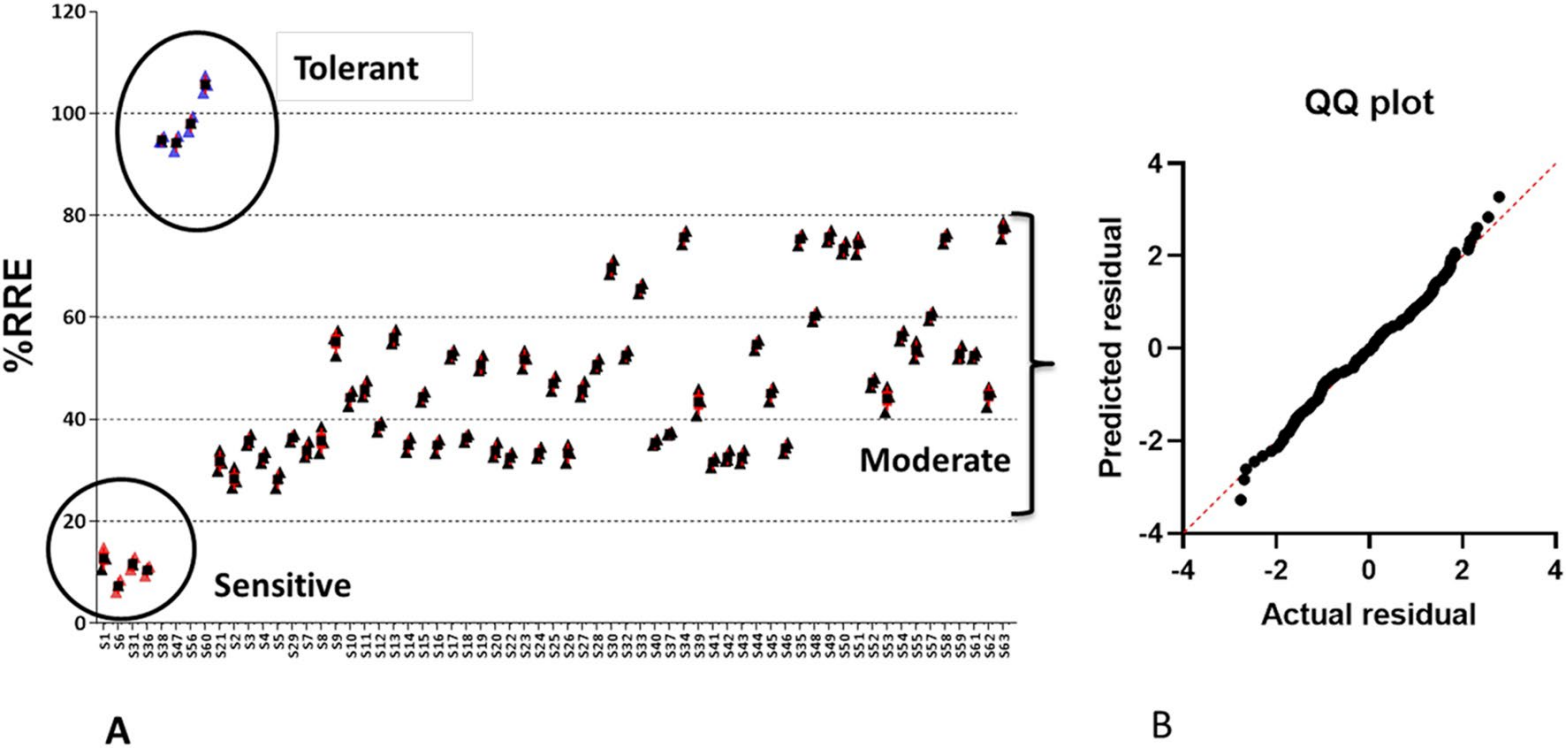
(**A**) RRE (relative root elongation) of 63 different rice genotypes under aluminum (200 μM) stress for 24 h (means ± *SE*, *n* = 10). (Details of S1 to S63 is given in Supplementary Table S1) (**B**) One way ANOVA: The QQ plot suggest normal distribution of the dependent variable (RRE).

The RRE results showed that root growth of S1, S6, S31, and S36 was significantly inhibited after 24-h exposure to 200 μM Al, with their RRE being nearly 7.35% of the control. The highest RRE (71.47%) was found in Motodhan. According to the RRE cluster analysis, the four cultivars Motodhan, Vietnam-1, Yimyu and N-861 were tolerant. The Al tolerance values of Lespah, RCPL-13, VL-31,329 and UPR 2919-141-1 were low and the genotype was found to be sensitive to Al (Fig. 4). The results showed that the Al tolerance of Motodhan was the highest among the 63 rice genotypes/varieties.

### Aluminium toxicity and root water-relations

**Figure 5:**
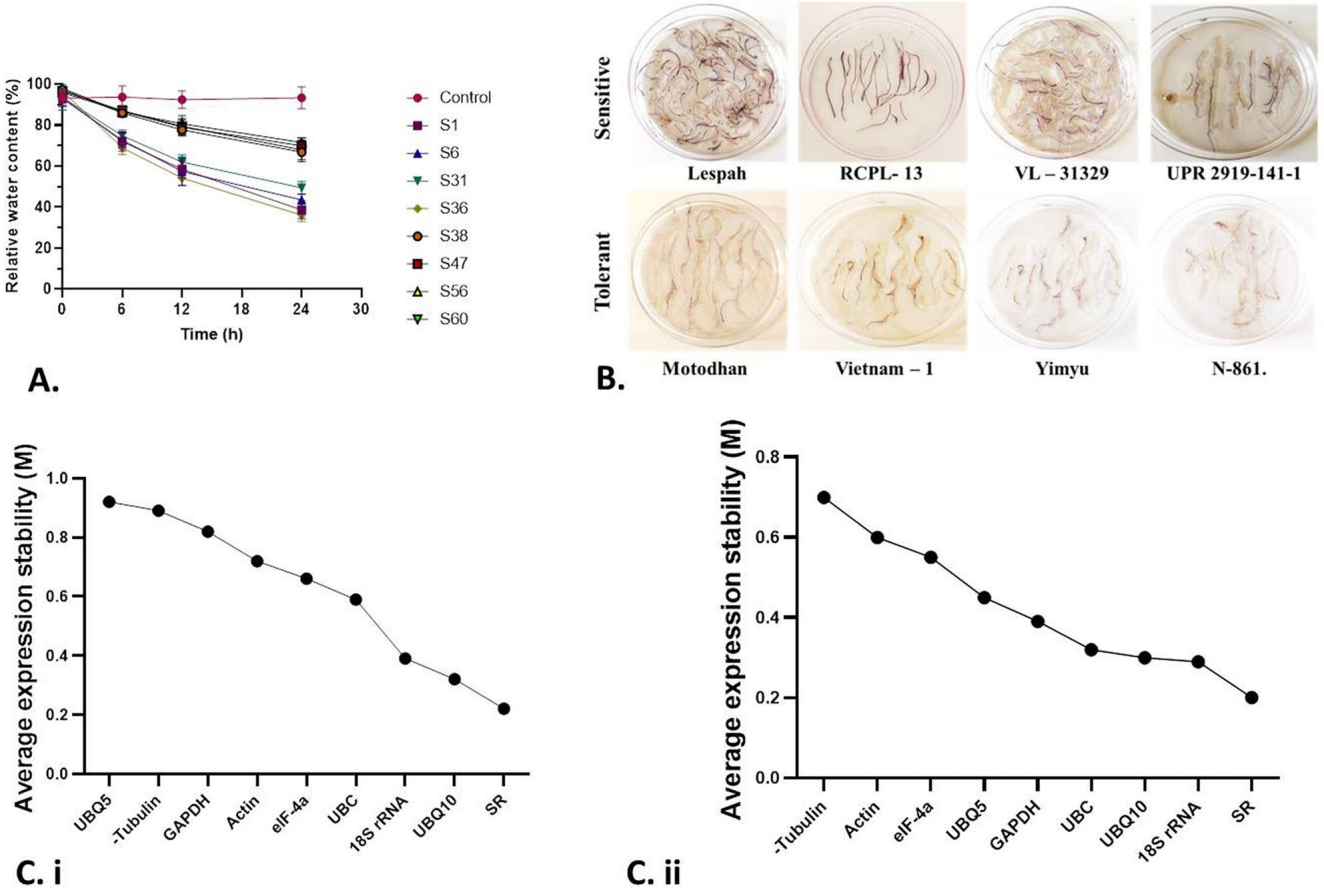
(**A**) Relative water content estimates during the imbibition period for hydrated and water-stressed roots of Al-resistant cultivars (ARC) and al-sensitive cultivars (ASC). Vertical lines indicate the mean standard error (Details of the S1, S6, S31, S36, S38, S47, S56, S60 given in Supplementary Table S1); (**B**) Hematoxylin staining of roots of sensitive and tolerant rice genotype at 200 µM Al-concentration; (**C**) Expression stability and ranking of endogenous control genes as calculated by geNORM in all eight cDNA samples: (**C.i**) control & (**C.ii**) 200 μM Al treated rice genotype root sample. A lower value of average expression stability, M, indicates more stable expression.

To identify probable relation between aluminium toxicity and root water relation, we calculated relative root water content in the roots of each treated line. Motodhan, Vietnam-1, Yimyu, and N-861 showed the highest RRWC values, which were almost as high as those of the control group, indicating that they did not respond to Al stress (Fig. 5A). In contrast, genotypes such as Lespah, RCPL- 13, VL-31,329, UPR 2919-141-1 showed a significant decrease in RRWC indicating that these plants cannot maintain the turgor pressure of their roots and the root elongation in the presence of Al.

### Histo-chemical detection of hematin

Hematoxylin assay clearly showed marked colour variations in the roots of rice genotype between the tolerant and sensitive Al-treated plants (Fig. 5B). While Al-toxicity sensitive rice genotype viz*.,* Lespah**, **RCPL- 13, VL–31,329 and UPR 2919-141-1 showed dark blue staining suggesting higher accumulation of Al in the roots; in contrast rice genotype viz*.,* Motodhan, Vietnam-1, Yimyu and N-861 showed negligible staining indicating Al-toxicity tolerant genotype/varieties accumulated lesser Al in the roots.

### Identification of Al-resistant and Al-sensitive rice cultivars for gene expression study

Based on root growth and biomass, root tolerance index (RTI), and relative root elongation (RRE%) upon exposure to 200 μM AlCl3, Al stress-resistant and susceptible genotypes were identified from 63 rice genotypes. Four highly tolerant Al-resistant cultivars (ARC) Motodhan, Vietnam-1, Yimyu, N-861 and four significantly susceptible Al-sensitive cultivars (ASC) Lespah, RCPL- 13, VL-31,329, UPR 2919-141-1 were further identified for screening the molecular transcriptional responses underlying the differences in Al resistance using Al-sensitive genes with significant effect. These genes were analysed for the above eight genotypes by quantitative real-time qRT-PCR. However, before doing so, we identified an appropriate endogenous control gene (ECG) to be considered as the best reference gene. These genes were selected from four different domains, such as biotic stress, abiotic stress, developmental stages, and target tissue, based on the previously published research article on Al toxicity in rice. Previously published primers (Supplemental Table: 1) for *GAPDH, tubulin, eIF-4α, UBQ5, 18S rRNA, actin, UBQ10,* and *SR* were verified by qRT-PCR using cDNAs synthesised from the roots of the above rice genotype.

### Effectiveness and analysis of stable expression of endogenous control genes

The identification and selection of a stable ECG is the crucial step in any RT-PCR analysis. ECGs that regulate various metabolic functions showed variations in expression levels in the presence of Al stress. We examined the expression of several endogenous genes under control (0 µM Al) and 200 µM Al treatment conditions for Motodhan rice, which are important for normal cellular homeostasis, in search of a suitable endogenous control. The amplification efficiencies of the ECGs were calculated individually using the logarithm (log) of the cDNA dilutions for the root samples. The most appropriate dilution for sample amplification was 1:25, which was subsequently used for target gene validation. The genes studied were *actin, tubulin, UBQ5, GAPDH, eIF-4α, 18S rRNA, UBQ10,* and *SR* (sulfite reductase). Amplification curves for each gene were generated and grouped across all root tissue samples, and the cycle threshold (CT) was set at a fluorescence threshold of 0.2. The geNORM v3.4 software was used to analyse the expression stability of the tested genes in different tissue samples and classify them accordingly. geNORM is a statistical algorithm that determines the gene stability measure (M) of all tested genes based on geometric averaging of multiple control genes and signifies the pairwise deviation of a gene from all other control genes in a given group of samples. It is based on the principle that the expression ratio of two ideal internal control genes is identical in all samples, regardless of the experimental conditions and cell type. The genes with the lowest M values have the most stable expression. When all eight root tissue samples were considered together, the average expression stability value (M) of *UBQ10* and *SR* was lowest and that of *UBQ5* was highest (Fig. 5C). The results remained very similar when the M value was measured at 200 μM Al stress, with the lowest value for SR and 18S rRNA. At 200 μM Al treatment, the M value was lowest for the *18S* *rRNA* and *SR* genes, followed by *UBQ10*, *UBC*, and *GAPDH*, although the difference between the M values was not very significant (Fig. 5C). A similar pattern of results showing SR as the most stable ECG was also obtained with RefFinder. The qRT-PCR analysis showed that the expression of most endogenous control genes (ECGs) changed under Al toxicity compared with untreated controls (Fig. 5C). While the expression of *actin* and *18S rRNA *increased significantly with increasing Al exposure time from 0 to 24 h at 200 µM AlCl_3_, the expression of *tubulin, UBQ5, UBQ10* and *GAPDH* decreased significantly with increasing Al treatment time.

In contrast, no significant difference in *SR* expression was observed with prolonged Al exposure (supplementary Fig. 2). Similarly, relative expression of ECGs between control and 200 µM Al treatment showed that the *18S rRNA* gene and *UBQ10* genes were comparatively persistent under Al stress. The *SR* gene was consistently expressed during Al treatment in the ASC and ARC rice genotypes. Among the candidate genes examined, only the *SR* gene showed stable expression over time (Fig. 5C). The gene *SR* was then used to analyse a set of Al-responsive genes to initially find four Al-resistant rice cultivars (ARC). About 20 previously published Al-responsive genes were selected to check their expression in our rice lines. Genes were selected based on their published cDNA full-lengths and respective functionalities. All these genes were previously screened with rice cultivars for Al-responsiveness by Northern blotting and qRT-PCR.

### Comparative expression of Al responsive gene in Al-resistant rice cultivars (ARC) and Al-sensitive rice cultivars (ASC)

**Figure 6:**
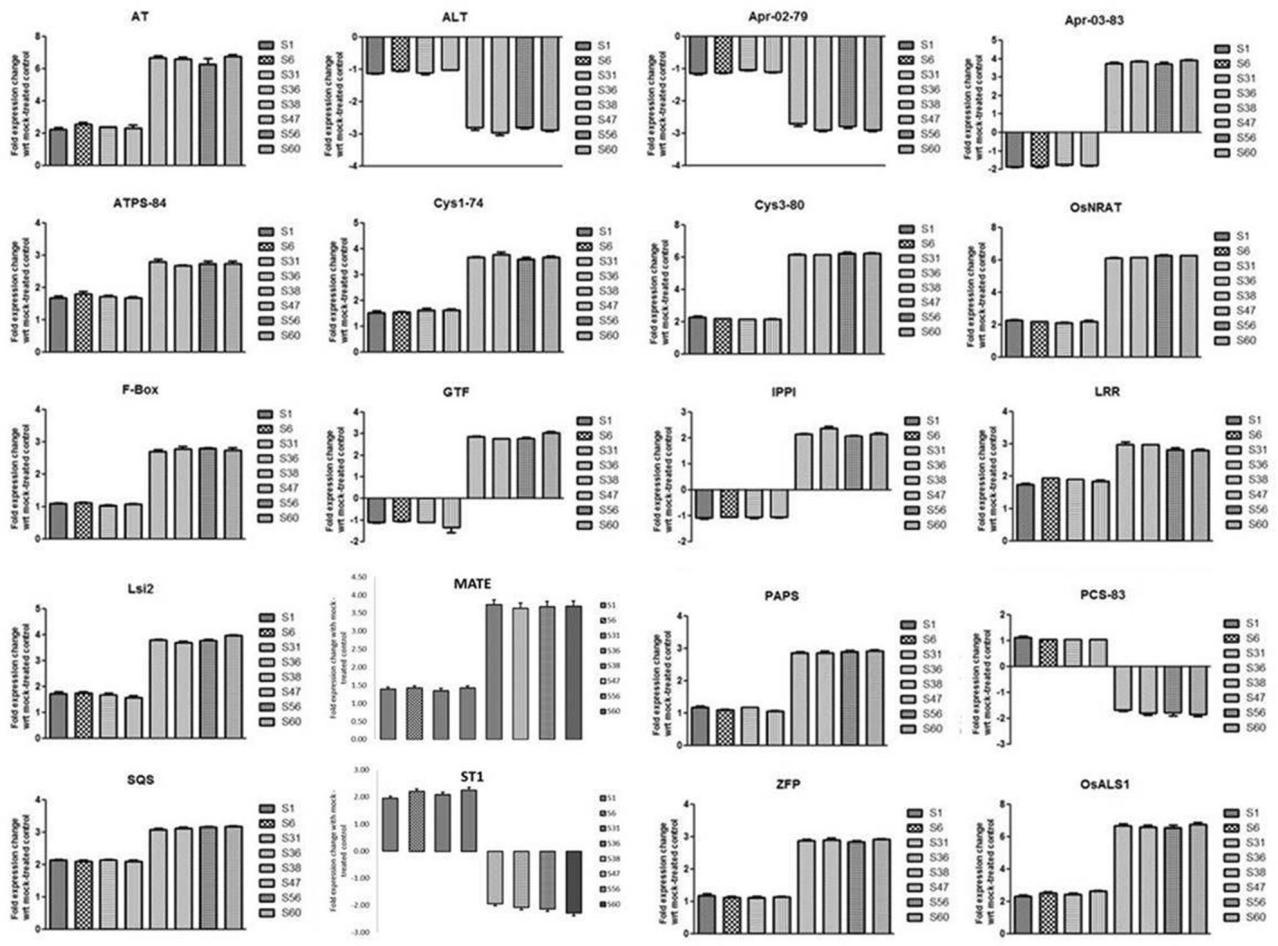
Al-induced fold expression changes (with mock-treated control) of Al-responsive genes viz., ALT, Alanine aminotransferase; AT, Putative anion transporter; ATPS, ATP sulfurylase; CYS1, an isogene of cysteine synthase; CYS3, an isogene of cysteine synthase; F-box,F-box domain protein; Apr-02, an isogene adenosine 5'- phosphosulphate reductase; Apr-03, an isogene adenosine 5'- phosphosulphate reductase; GTF, Putative glucosyltransferase; IPPI, Isopentenylpyrophosphate isomerase; LRR, Leucine-rich repeat family protein; Lsi2, Silicon efflux transporter; MATE, Multi-drug and toxin extrusion transporter; Nrat1, Nramp aluminum transporter 1; OsALS1, ABC transporter; PAPS, Purple acid phosphatase; PCS, phytochelatin synthase; SQS, Squalene synthase; ST1, Putative sulphate transporter 1; ZFP, Zinc finger protein-like in rice genotype (S1,S6, S31, S38, S47, S56 and S60).

Al-resistant rice cultivars (ARC) S38, S47, S56, and S60 were used to screen a number of potentially important target genes for Al response. The relative expression of these genes was then compared with that of Al-sensitive rice cultivars (ASC) S1, S6, S31, and S36. The fold- change values of each gene are shown in supplemental Table 3. After Al treatment, thirteen genes were transcriptionally up-regulated in all cultivars. For example, AT (anion transporter), *ATPS* (ATP sulfurylase), *F-box, Lsi2* (silicon efflux transporter), *SQS* (squalene synthase), *MATE* (multidrug and toxic compound extrusion), *PAPS* (purple acid phosphatase), *Cys 1, Cys-3, OsNrat1* (Nramp aluminium transporter 1), *LRR* (leucine-rich repeat family protein), *ZFP* (zinc finger protein), and *OsALS1* (amyotrophic lateral sclerosis) gene showed almost 3, 2, 3, 2, 2.7, 2.5, 1.3, 3, 3, 1.2, 3, and threefold higher expressions in ARCs compared with ASCs, respectively (Fig. 6). In contrast, Al treatment transcriptionally suppressed a number of key regulatory genes that involved in cellular mechanisms genes. For example, *ALT (*Alanine aminotransferase), *APR2* (5′-adenosine phosphosulphate reductase) *ST1* (sulphate transporter 1) and *PCS* (phytochelatin synthase) were significantly down-regulated by Al toxicity in both ARC as compared to ASC. Additionally, while *GTF* (putative glucosyltransferase) and *IPPI* (Isopentenylpyrophosphate isomerase) showed negligible expression in ASC, they showed very high expression in ARC.

## Discussion

Cereal crops such as rice, maize, and wheat are an ideal model crop to study the mechanism of Al toxicity tolerance due to their diverse genetic resources and an active and large research community related to agriculture^12^. In addition, work on one cereal species can quickly impact the entire family. Although rice is known as an Al toxicity tolerant crop, the toxic effect of Al is influenced by the concentration of the metal ion. It varies depending on the species, including genotype within the same species, physiological age, growing environment, and period of Al exposure. NE India is naturally one of the most acidic land significantly affects the rice varieties grown in this region and thus provides a useful study site for Al toxicity in rice. In our study, sixty-three (63) different rice lines (genotypes/released varieties) growing in NE region of India were selected to gain a better understanding of root growth and differentially expressed genes upon exposure to higher concentration of aluminum chloride. The different rice lines tested for germination using the Hoagland nutrient solution with or without Al added (no aluminum) showed significant differences in sensitivity to Al at the germination stage. A major feature of Al toxicity is the inhibition of root growth upon exposure to lethal concentrations of this metal in soil, and this consequence has also been observed in many plant species in recent decades^13^. However, our results clearly showed that 200 μM Al treatment did not affect root growth of some rice genotypes (Motodhan, Vietnam-1, Yimyu, and N-861), indicating a possible Al toxicity tolerance mechanism in these lines.

Seedling root length is often used as a selection criterion for Al toxicity tolerance in crops such as rice, sorghum, soybeans, or wheat^14^. According to the calculations of RTI in our study, Motodhan seedling (upland rice) was the most tolerant genotype, followed by Vietnam-1, Yimyu, and N-861 with RTI values above 0.987. This result indicates that an Al concentration that is cytotoxic to the susceptible rice genotype can stimulate root growth of the tolerant genotype. This phenomenon has also been observed previously in rice^15^. Rout and Das^16^ reported that the rice genotype with RTI values greater than 0.90 can be classified as tolerant. Wu et al*.*^15^ also proposed a similar classification for tolerance to Al toxicity in rice. However, the relation between the Al toxicity and water uptake is still poorly understood. Water uptake into root cells is usually controlled by the water potential gradient, which acts as a driving force for water uptake and the buildup of turgor-a prerequisite for cell expansion. According to recent studies, Al-induced root growth inhibition does not directly explain low water uptake as hydraulic and hydric parameters are affected prior to root growth inhibition^17^. Therefore, high abscisic acid content (ABA) and low hydraulic conductivity of roots could explain the low hydration of leaves in Al-exposed plants. Moreover, Al-induced stomatal closure is usually associated with low root hydraulic conductivity and high ABA accumulation^18^.

RRWC and RRE % showed that Motodhan, Vietnam-1, Yimyu and N-861 were more tolerant compared to the other genotypes. Therefore, we classified the studied genotypes into three groups based on the above physiological parameters: tolerant, moderately tolerant, and susceptible (Table 1). In addition, the tolerant and susceptible genotypes were considered for the study of relative gene expression.

**Table 1:** Classification of 63 rice genotype into Al-tolerant, moderately tolerant and susceptible based on Root tolerance Index (RTI) values.

RTI value	Class	Rice varieties	No. of genotype
≥ 0.90	Tolerant	MOTODHAN, VIETNAM-1, YIMYU, N-861	4
0.65–0.90	Moderately tolerant	CHANKIMASO, ANJALI, MOIRAMSBHI, COL- 4, CHING 8. IR 1552, SHAKU 10. IORO, SKAU-390, VR-14, VL31329, VL 31,331, UPR 2919-14-1–1, UPR 2992-17-3-1, PANCOAS, ASUKNI MAGHOWA, ASSAM, LONGPA TSUK, MICHIYING, BANG NAYK, YIMYA MAPOK, SANG CHANG, MEYISAO, NUNG KHUM, AKIYIUTI ASHE, BHALUM -2, BHALUM -3, CHING MOIRAMSBHI, KHASHA, KALOJEERA, KHOUGJAI PHOU, POSIMOT, ZAM, SANRI FIIRII, TSAMU FIIRII, BHALUM – 1, MERANGKONG, KONPEMO, CHIHG, BHALUM – 4, LIKHAMO, DHAO TIPNUAKULON, , AAHA, SILKY RICE, KENASU KEDOWA, KOYABO, MANGE, TSAKNAK, EPYO, HAHSHO, MOMCHING, 58. IR 72, SATABDI, MTU – 7029, GOBINDOBHOG, IDAO	55
0.20–0.64	Susceptible	LESPAH, RCPL- 13, VL-31,329, UPR 2919-141-1	4

Most of the genes that regulate various metabolic functions are often considered as endogenous control genes (ECGs)^19^. Our study on the expression stability of ECGs under control and Al treatment condition showed Actin, Tubulin, eIF-4α, UBQ5, and GAPDH significantly varied in both Al concentration and time frame which is in accordance with the report of Jain et al.^20^. On the other hand 18S rRNA gene and UBQ10 genes were comparatively persistent under Al stress. Similar results were observed by other researchers^20,21^ who considered that 18S rRNA is the most stable endogenous control gene under Al toxicity. However, 18S rRNA has disadvantages to be treated as the finest endogenous control^22^ since the 18S rRNA genes are tremendously abundant as compared with those of most of the target genes, tough to regulate optimal template concentrations and most importantly ribosomal RNAs commonly lack poly-adenylated tails, such that oligo-dT primers can’t be used for the cDNA synthesis. On the other hand, SR (Sulfite reductase) gene was expressed constantly in ASC and ARC rice genotype during the Al challenge, which was also reported by Zhang^23^. According to the general acceptance criteria, the ideal endogenous control gene is stably expressed (or, at least, only slightly varying in the expression) among the sets of samples investigated and has an expression level compared to the target gene^24^. SR shows a stable expression under varying Al stress as well as at different times, it was therefore selected as the endogenous control in our gene expression study. SR has advantages over other traditional reference genes used in many previous studies^22^. Our results are consistent with the previously reported data further validating the reliability of the qRT-PCR analysis using SR as an EC gene^20,21,23,25^.

While the physiological mechanism of Al toxicity is relatively well documented, a detailed molecular mechanism for Al toxicity tolerance in rice still requires additional research^26^. Recently, several Al-responsive genes such as *STAR1, STAR2, Nrat 1,* or *ALS 1* have been characterized; however, the expression of these genes in response to Al treatment is genotype-specific. Moreover, the available gene expression studies exploring Al toxicity in genotypes of NE India are limited to 2–4 genotypes. Thus, a clear picture of Al toxicity in rice from NE India is still lacking. Therefore, to achieve a better mechanistic understanding, there is an urgent need to study genetic variability and molecular characterization of several genes for Al specificity. Based on the available database, we selected twenty putative Al-responsive genes and enumerated the comparative expression profile between ARC and ASC to decipher potent genes that can be developed as molecular markers for Al sensitivity in rice. After Al treatment, thirteen genes were up-regulated in all cultivars studied. For example, Al-induced expression of *LRR* and *Lsi2* was more pronounced in the resistant cultivar ARC than in the susceptible ASC. Both *AT* and *MATE* were previously identified as Al- up-regulated citrate transporters involved in citrate secretion. The results are consistent with those previously published by Zhang et al*.*^23^ and Chandran et al.^25^ confirming the consistency of the quantitative RT-PCR analysis using SR as the control gene. Both *Nrat1* and *OsALS1* expression are induced by Al and regulated by the transcription factor ART1^27^. Overexpression of Nrat1 increases sensitivity to Al, as *Os*ALS1 can then become a limiting factor for Al detoxification.

There are several Al-upregulated genes, which are related to the sulphur acquisition and metabolism^23^. Our study clearly revealed that upon Al treatment, expression of *ATPS* and isoforms for cysteine synthase (*CYS1* and *CYS3)* were most pronounced at 24 h post-treatment. The *APR3* gene was also induced with Al treatment. Some members of ZFP family reported to be induced by abiotic stresses^28^. To check the role of *ZFP* in aluminium toxicity tolerance, a new primer pair was prepared from the RING-variant domain which is a zinc-finger-like motif found in a number of cellular and viral proteins. In the present experiment, *ZFP, IPPI*, *SQS* and *GGPPS* were also up-regulated with an aluminium dose response. *IPPI*, *SQS* and *GGPPS* genes are known to be involved in the functional biosynthetic pathway of terpenoids and carotenoids^29^. Carotenoid plays a crucial role to combat oxidative stress^28^ reminiscent of Al toxicity^30^. In addition, the exudation of some phenolic compounds including terpenoids is reportedly involved with Al resistance in plants^31^.

Our study showed that Al treatment transcriptionally suppressed the expression of a number of genes. For example, *GTF, PCS, ST1, ALT,* and *APR2* were significantly downregulated by Al toxicity. GTF is known to catalyse the transfer of sugars to a wide range of acceptor molecules. Therefore, expression of GTF in the presence of reduced Al could ameliorate oxidative stress associated with secondary metabolism, consistent with evidence that Al toxicity is associated with reactive oxygen species production^30^. *APR2* and *PCS* were strongly suppressed by Al stress. A number of Al-inhibited genes are associated with cell metabolism. *PCS* is an Al-inhibited gene involved in sulphur acquisition and metabolism. The dominance of ALT and ST1 may have disrupted the basic metabolism of C, N, and S, ultimately leading to stunted root growth in Al-sensitive rice lines compared with Al toxicity tolerant rice lines.

## Conclusion

The rice genotypes grown in the North-east India are known to possess various agronomical important traits like biotic and abiotic stress tolerance, unique grain and cooking quality. The effect of Al-toxicity tolerance studied among 63 genotypes grown in North-east India revealed Motodhan, Vietnam-1, Yimyu and N-861 as most Al-toxicity tolerant, while Lespah, RCPL-13, VL-31329 and UPR 2919-141-1 as Al-sensitive genotypes. The four contrasting rice genotype with different Al sensitivities and the identified Al-regulated gene expression profiles between the cultivars will provide a good foundation to elucidate molecular mechanisms that are responsible for the sensitivity differences. The transcriptional regulation of these genes in responding to Al stress in this particular Al-resistant cultivar may provide important clues leading to the final identifications of genes that may be utilized by the plant breeders for the development of Al-tolerant rice crop for highly acidic soil by conventional breeding /MAS breeding/Genetic engineering.

## Materials and methods

### Plant material

A set of 63 Rice (*Oryza sativ*a L.) genotype (upland and lowland rice genotype) grown in different parts of NEH, India were used for this study (Table 1). Seeds used in this study were obtained from ICAR Research Complex for NEH Region, Umiam, Meghalaya.

### Nutrient culture and aluminum treatment

Healthy grains from each rice genotype were surface sterilized using 70% ethanol for 2 min followed by 0.1% (v/v) HgCl_2_ for 7 min, and then rinsed with autoclaved distilled water. The seeds were then soaked in water for 24 h, followed by germination in a petridish on moistened filter paper for 72 h at 28–31 ± °C in the dark. After the incubation period, germinated seedlings of visually uniform shoot and root lengths were transferred to a plastic container (45 cm × 32 cm × 17 cm) containing water supported with perforated styrofoam sheets (10 lines × 13 rows) and nylon net bottom. Five days later, all seedlings were exposed to half-strength Hoagland nutrient solution^32^. The pH of the nutrient solution was maintained at 4.5 to mimic the acid soil pH of the North-Eastern hills of India. The plants were grown under white light for 15 days followed by a 16/8 h photoperiod with a light intensity of 230 μmol m^−2^ s^−1^. The solution was renewed daily to preserve healthy growth conditions and avoid the precipitation of chemicals. Hydroponics was continuously aerated for a proper supply of oxygen to the root system. After 15 days of photoperiod treatment, rice seedlings were pre-treated with 500 μM CaCl_2_ (pH 4.5) for a day to minimize the phytotoxic effect of aluminium on certain physiological processes, such as plant growth, chlorophyll, membrane lipid content and the antioxidant defence system. At this stage, the root length (from root-shoot junction to the tip) of the seedlings was measured and used as the initial length. Aluminium treatment was done by adding AlCl_3 _(Sigma) to a fresh pre-treatment solution (200 μM) containing 500 μM CaCl_2_ (pH 4.2 adjusted with HCL and NaOH) for 3 days. Variations in root growth of treated rice genotype were observed after every 12 h. However, all our experiments were standardised at 24 h. No addition of AlCl_3_ was used in the control. Free Al3 + activities in treatment solutions were calculated using GEOCHEM-PC speciation software.

As rice is a comparatively aluminium toxicity tolerant crop among all the cereal crops, one separate experiment was also conducted to determine the optimal Al concentration for screening all the rice genotype used here. An assay was conducted at the germination stage using rice seeds of all 63 rice (5 seeds of each line) lines. Seeds were surface sterilized and cultured on germination paper soaked in six different concentrations (50, 100, 150, 200 and 250 μM) of AlCl_3_ and the pH of each solution was adjusted to 4.2 with 0.2 N H_2_SO_4_ in order to find the optimum Al concentration for follow up experiments. The same set of seeds also germinated under the unstressed conditions with half-strength Hoagland solution having pH adjusted to 4.2 and was used as control. Seeds were allowed to germinate for 4 days at 28–31 ± °C in the dark and then were transferred to light (16/8 h photoperiod with a light intensity of 230 μmol m^ − 2^ s^ − 1^) and grown for another week. Seeds were considered to be germinated when radicles and plumules could be clearly distinguished. Cultures were soaked every day with a half-strength Hoagland solution containing Aluminum chloride at pH 4.5. The rate of germination and root growth was visually observed^33^.

#### Growth and biomass analysis

Growth analysis was performed by measuring the root length; fresh and dry root weight of treated and control seedlings after 3 days of treatment. 12 plants of each genotype were randomly selected and the length of the root was measured. Root tissues were dried at 60 °C for 2–3 days and weighed to determine the dry biomass. The fresh weights, dry weights and root lengths were used to estimate the relative root length (RRL), relative tolerance index (RTI) and relative root reduction (RRR%) after Al^3+^ treatment. Each experiment was repeated thrice.

As a relative measure, gain or loss of fresh biomass (Δm) was defined as:

$$\Delta {\text{m}} \, = {\text{m}}_{{{\text{after}}}} {-} {\text{m}}_{{{\text{before}}}}$$

with m_after_ representing the plant weight in g of the whole plant at the end of the experiment, and m_before_ the plant mass before the experiment.

### Measurement of root tolerance index (RTI)

Root tolerance index (RTI) was calculated as the maximum root length in Al stressed culture divided by the maximum root length in control^34^. Based on RTI values, the rice lines have been classified into three groups—(i) Highly Tolerant, (ii) Moderately tolerant and (iii) Susceptible as ≥ 0.90, 0.65–0.90and 0.20–0.64 respectively.

### Measurement of relative root elongation (RRE)

Relative Root Elongation (RRE) was used for estimating Al tolerance in 63 rice lines. The formula is shown as below:

$${\text{RRE }}\left( \% \right) \, = \, \left( {{\text{RL}}_{{{\text{Al}} + }} {-}{\text{ RL}}_{{{\text{Al}} - 0}} } \right) \, / \, \left( {{\text{RL}}_{{{\text{CK}} + }} {-}{\text{ RL}}_{{{\text{CK}} - 0}} } \right) \, \times { 1}00$$

where, RL_Al+_ and RL_Al-0_ are the lengths of the longest root after and before Al treatment, respectively, and RL_CK_ + and RL_CK-0_ are the lengths of the longest root of the control after and before treatment, respectively^35^.

### Measurement of root relative water content (RRWC)

Root Relative Water Content (RRWC) was determined by weighing the root and floating it on water (deionized water) for 6 h at constant temperature in dim light. When root became fully pompous, it was re-weighed, dried and again weight was measured. RRWC was calculated using the formula:

$${\text{RRWC }} = \, \left( {{\text{FW }} - {\text{ DW}}} \right)/\left( {{\text{PW }} - {\text{ DW}}} \right) \, \times { 1}00$$

where, FW = Fresh weight, DW = Dry weight and PW = Pompous weight^36^.

### Histochemical study of Al-toxicity by hematoxylin assay

Roots from the Al-treated rice genotype were collected and stained with hematoxylin according to the method described by Yu et al*.*^37^. Briefy, roots from each genotype were treated with a solution mixture of 0.2% heamatoxylin and 0.02% KI followed by rinsing with de-ionized water.

### RNA extraction, cDNA synthesis and expression profile of endogenous control gene (ECG) and target genes

Total RNA was isolated from 50 mg fresh root tissues of eight (8) rice lines Lespah, RCPL- 13, VL-31,329, UPR 2919-141-1 (Al toxicity susceptible rice lines) and Motodhan, Vietnam-1, Yimyu, N-861 (Al toxicity tolerant rice lines) using Trizin (GCC Biotech, India) as per manufacturer’s protocol. RNA quality and quantity were assessed by denaturing RNA agarose gel electrophoresis^38^ and spectrophotometric detection at 260 nm and 230 nm using Chemi Doc system (Bio-Rad). cDNA synthesis was carried out with oligo-(dT) primer using R2D cDNA Synthesis Kit (GCC Biotech, India).

For endogenous control genes (ECG), we selected eight primer pairs (supplementary table S2) widely reported in literature. In a wide range of studies with rice these were used as internal control in real-time quantitative PCR analysis. As these genes play role in both biotic and abiotic stresses and show overlapping action, all of them were tested to identify the best ECG for the current experiment (Al stress) with the selected set of rice lines.

### Specification and analysis of stability expression of reference genes

The PCR effectiveness was assessed from four serial dilutions of cDNA (1:1, 1:5, 1:25, and 1:125) to generate the standard slope for each pair of primer tested. The value of E was estimated by the equation E = 10^(−1/slope)^^39^, and values of effectiveness between 1.74 and 1.9 were considered as acceptable for reference genes.

Performance of each reference gene was classified and determined by the average Ct values for each test sample obtained by each reaction cycle in real-time RT-qPCR. Data analysis was subjected to results of variance, using the Statistical Analysis System—GraphPad Prism7 software.

The stable reference genes were considered on the basis of lower standard deviation and coefficient of variation using geNorm v3.4 (https://genorm.cmgg.be/). The detailed calculation is described in Chen et al*.*^40^. We further confirmed the stability of ECGs using ref Finder. The mean Ct value of each sample for each primer was used as input data on the analysis, and the Ct value belonging to the crop and the weed were analysed altogether. It assigns an appropriate weight to an individual gene and calculated the geometric mean of their weights for the overall final ranking.

### Optimization of primer anneal using semi-quantitative PCR

We chose a two-step qRT-PCR protocol where reverse transcription and PCR-mediated cDNA amplification are carried out in subsequent steps in separate tubes. The two-step protocol which diminishes unwanted primer dimer formation is preferred when SYBR Green is used as a detection dye. The reverse transcriptase reaction was primed with oligo-(dT) instead of random-sequence primers because the latter preferentially selects for more abundant mRNA species and not for transcripts of weakly expressed genes.

Ideal PCR cycle for each and every gene was optimized during semi-quantitative PCR analysis. The optimum cycles need ranged within the log-linear phase of the amplification cycle. PCR reactions were conducted at 94° for 3 min, 30 cycles of 94° for 30 s, and 55°–60° for 30–40 s (depending on primer) and 72° for 40 s, and a final extension step at 72° for 5 min. The PCR was performed employing Simpli Amp Thermal Cycler (Invitrogen), and the PCR products were separated on 1% (w/v) agarose gels and visualized by EtBr staining. All PCRs were repeated twice using the same cDNA sample. Quantifications of PCR products were made by densitometry analysis using Image Lab™ Software (Bio-Rad). The experimental reproducibility was assessed by using different RNA preparations from the same experiment and two different PCR cycles.

To verify semi-quantitative PCR results, seven already reported Al-tolerance genes were used (Supplementary Table S2). The genes were chosen on the basis of published data and respective transcriptional functionalities like cell division, growth and development, apoptosis and, physiological processes such as glycolysis, metabolism, etc. To validate the semi-quantitative PCR results twenty newly designed real time quantitative PCR based primers from Al-responsive genes were prepared (Supplementary Table S2). The Primer Express Software v3.0.1 (Applied Biosystems™) was used in order to design primers for amplification of Al-responsive genes. Due to the presence of experiments with stress by aluminum excess, conditions of aluminum deficiency were used in the search of these genes. We also selected a few already published primers from previously identified Al responsive genes for reliability of the semi-quantitative PCR analysis.

### Quantitative real-time PCR (qRT-PCR) analysis

The PCR reactions consisted of 10 µl of 2X H-effqPCR master Mix, Rox (GCC Biotech, India), 200 nM of forward and reverse primers, and 2 µl of 1:20-diluted template cDNA in a total volume of 20 µl. The PCRs with no template controls were also performed for each primer pair. The real-time PCR was performed employing a Quant Studio 3 Real-Time PCR System and Quant Studio™ Design and Analysis Software (Invitrogen). All the PCRs were performed under the following conditions: 1 min at 95°, and 30 cycles of 95° for 30 s, 58° for 20 s and 72° for 15 s in 96-well optical reaction plates. The specificity of amplicon was verified by melting curve analysis (70–95°) after 35 cycles and agarose gel electrophoresis. Three technical replicates of each sample were used for the real-time PCR analysis.

### Validation of reference genes and specificity of target genes

In order to ensure the reliability of the potential reference gene, the expression profile of twenty genes were measured and normalized with the most stable reference gene as determined by geNORM v3.4, RefFinder and the analysis of variance. The amplification conditions by real-time qRT-PCR were the same as mentioned above. The relative expression of data were calculated using the formula QR = 2^−(ΔΔCT)^^41^.

### Statistical analysis

The experimental plan used was complete randomized block design with 63 lines per replication and two replications, in a total of 126 experimental units; each unit consisting of three seedlings. Statistical analyses of data are shown with absolute values, using Al concentration and cultivar as variables. The data were subjected to standard statistical methods of analysis of variance (ANOVA) and significant differences between mean values of three independent experiments repeated, were determined by Bonferronianalysis *P* ≤ 0.05 was considered to show statistical significance.

## Supplementary Information

Supplementary Figures.

Supplementary Tables.

Supplementary Table 3.

## Data Availability

All data supporting the findings of this study are available within the paper and within its supplementary materials.
