# Supplementary material for: Identification and expression pattern of aluminium-responsive genes in roots of rice genotype with reference to Al-sensitivity
Source: Sci Rep. 2023 Jul 27;13:12184. doi: 10.1038/s41598-023-39238-8 (PMC10374657; doi:10.1038/s41598-023-39238-8)
Supplement: Supplementary file 1 — Supplementary Figures. [file 41598_2023_39238_MOESM1_ESM.pptx]

## Slide 1
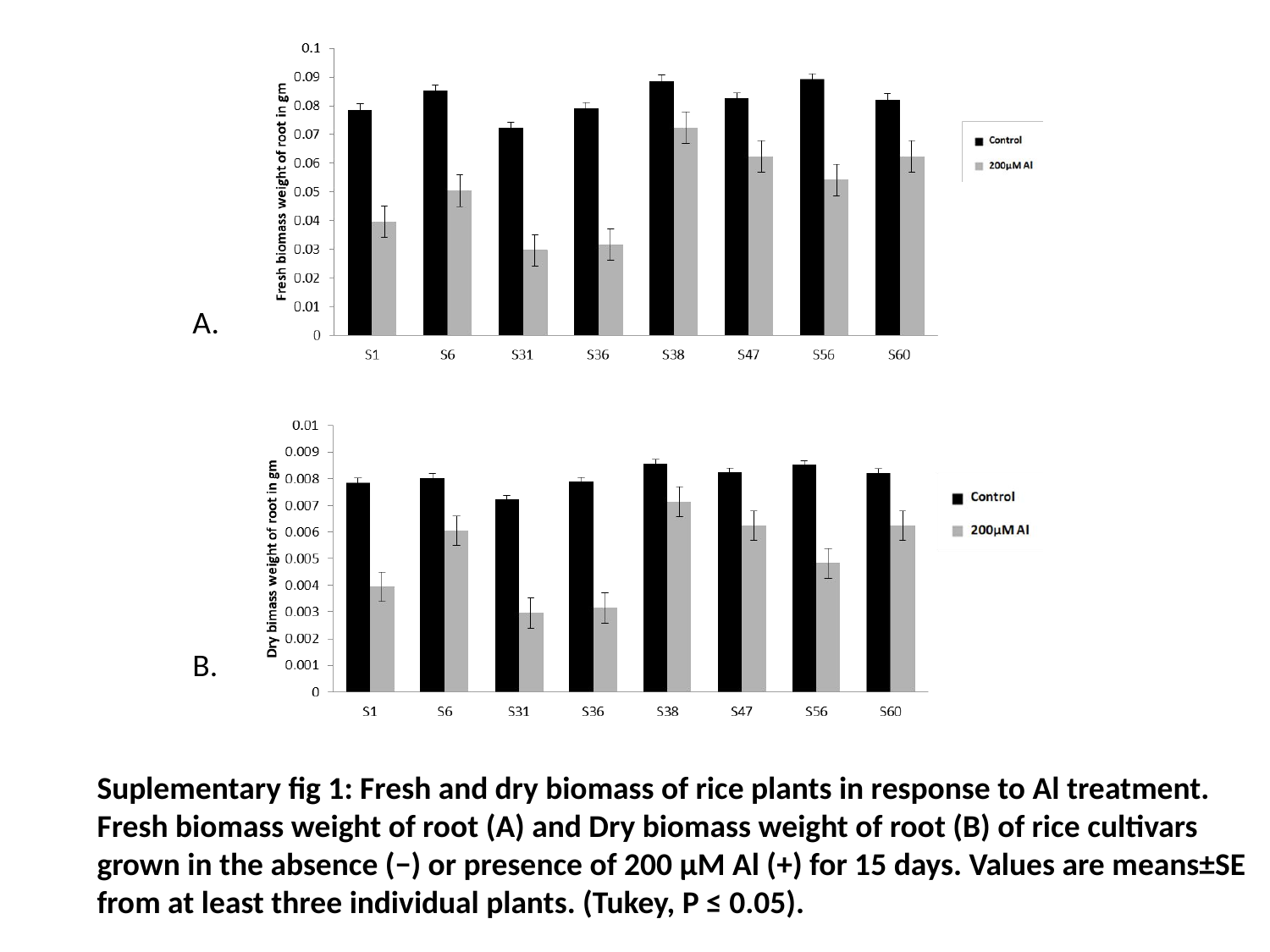

A.
B.
Suplementary fig 1: Fresh and dry biomass of rice plants in response to Al treatment. Fresh biomass weight of root (A) and Dry biomass weight of root (B) of rice cultivars grown in the absence (−) or presence of 200 μM Al (+) for 15 days. Values are means±SE from at least three individual plants. (Tukey, P ≤ 0.05).

## Slide 2
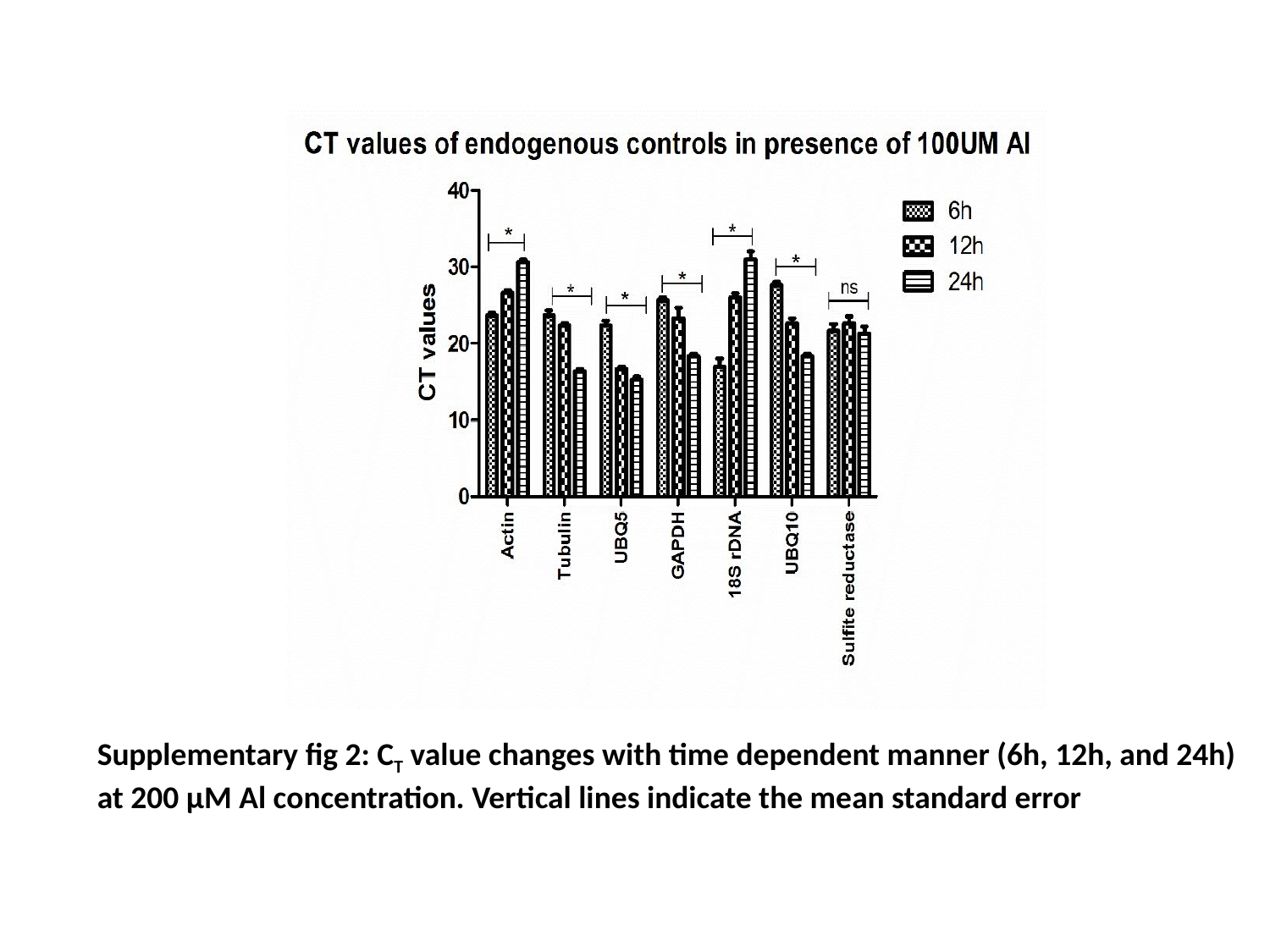

Supplementary fig 2: CT value changes with time dependent manner (6h, 12h, and 24h) at 200 μM Al concentration. Vertical lines indicate the mean standard error
