# Supplementary material for: Identification and expression pattern of aluminium-responsive genes in roots of rice genotype with reference to Al-sensitivity
Source: Sci Rep. 2023 Jul 27;13:12184. doi: 10.1038/s41598-023-39238-8 (PMC10374657; doi:10.1038/s41598-023-39238-8)
Supplement: Supplementary file 2 — Supplementary Tables. [file 41598_2023_39238_MOESM2_ESM.pdf]

Supplementary table 1: Rice genotypes used in the present study

Sl. No.	Name	CODE NAME	Germplasm/variety
1	LESPAH	S1	Germplasm
2	RCPL-13	S2	Released Variety
3	ANJALI	S3	Released Variety
4	MOIRAMSBHI	S4	Germplasm
5	COL- 4	S5	Germplasm
6	CHANKIMASO	S6	Germplasm
7	CHING	S7	Germplasm
8	IR 1552	S8	Released Variety
9	SHAKU	S9	Germplasm
10	IORO	S10	Germplasm
11	SKAO-390	S11	Germplasm
12	VR-14	S12	Released Variety
13	VIETNUM -3	S13	Germplasm
14	VL 31313	S14	Released Variety
15	ZAM	S15	Germplasm
16	UPR 2992-17-3-1	S16	Released Variety
17	PANCOAS	S17	Germplasm
18	ASUKNI MAGHOWA	S18	Germplasm
19	ASSAM	S19	Germplasm
20	LONGPA TSUK	S20	Germplasm
21	MICHIYING	S21	Germplasm
22	BANG NAYK	S22	Germplasm
23	YIMYA MAPOK	S23	Germplasm
24	SANG CHANG	S24	Germplasm

25	MEYISAO	S25	Germplasm
26	NUNG KHUM	S26	Germplasm
27	AKIYIUTI ASHE	S27	Germplasm
28	KASALATH	S28	Germplasm
29	BHALUM-3	S29	Released Variety
30	BHALUM -2,	S30	Germplasm
31	VL - 31329	S31	Germplasm
32	KHASHA	S32	Released Variety
33	KALOJEERA	S33	Released Variety
34	KHOUGJAI PHOU	S34	Germplasm
35	POSIMOT	S35	Germplasm
36	UPR -2919-14-1-1	S36	Released Variety
37	SANRI FIRRI	S37	Germplasm
38	MOTODHAN	S38	Germplasm
39	TSAMUM FIRRI	S39	Germplasm
40	BHALUM - 1	S40	Variety
41	MERANGKONG	S41	Germplasm
42	KONPEMO	S42	Germplasm
43	IR 72	S43	Germplasm
44	BHALUM - 4	S44	Released Variety
45	LIKHAMO	S45	Germplasm
46	DHAO TIPNUAKULON	S46	Germplasm
47	VIETNAM - 1	S47	Germplasm
48	AAHA	S48	Germplasm
49	SILKY RICE	S49	Germplasm
50	KENASU KEDOWA	S50	Germplasm
51	KOYABO	S51	Germplasm

52	MANGE	S52	Germplasm
53	TSAKNAK	S53	Germplasm
54	EPYO	S54	Germplasm
55	HAHSHO	S55	Germplasm
56	YIMYU	S56	Germplasm
57	MOMCHING	S57	Germplasm
58	BPT-5204	S58	Released Variety
59	SATABDI	S59	Released Variety
60	N-861	S60	Released Variety
61	SAHBHAGI DHAN	S61	Released Variety
62	GOBINDOBHOG	S62	Released Variety
63	IDAW	S63	Germplasm

Supplementary Table 2: Primer pairs used in the RT-PCR analysis

Gene	Functional Category	Genebank Accession	Forward Primer (5'-3')	Reverse Primer (5'-3')
GAPDH	glyceraldehyde phosphate dehydrogenase	AK064960	CAGGTCGGTCGGTTTTCTGA	ACGGTGTTGATCACCAGGTC
Actin	Actin-1	AK100267	CAGCCACACTGTCCCCATCTA	AGCAAGGTCGAGACGAAGGA
Tubulin	Tubulin	AK072502	GGACTATGAGGAAGTCGGCG	AGCAAGCAATGCACAGCAAA
Elf4	Early flowering gene	AK073620	CCTGTGTTGGTGGAACCTCT	TGGATCTTTGGTGGAAGGAG
UBC	Ubiquitin conjugating enzyme –E2	AK059694	CCGTTTGTAGAGCCATAATT GCA	AGGTTGCCTGAGTCACAGTT AAGTG
18S rRNA	18s ribosomal RNA	AK059783	GGCGGATGTTGCTTATAGGAC	AGACAAATCGCTCCACCAACT
SR	Sulphite reductase	AK073969	TACTTGGCTATGGATACGCT	ACAGGCACCTAATGATGAAC
UBQ5	Ubiquitin 5	AK061988	ACCACTTCGACCGCCACTACT	ACGCCTAAGCCTGCTGGTT
UBQ10	Ubiquitin 10	AK101547	TGGTCAGTAATCAGCCAGTT TGG	GCACCACAAATACTTGACGA ACAG
Apr-02	an isogene adenosine 5'- phosphosulphate reductase	XM_478340	GCAAGGACGTGTGGACCTTC	CCGATCGAGACGTACCCCTG
Apr-03	an isogene adenosine 5'- phosphosulphate reductase	AK059876	CCATCAATGGATCCAGCCGT	GCCGACCAGAATAGGCACTT
ALT	Alanine aminotransferase	AB007045	GGAGCATACAGCCACAGC	TGATAACTACCAAAGCCCTAAC
AT	Putative anion transporter	AK071480	CTGACAAATGCTGCCATCC	CAAGAGCCGCAGTGATGG
ATPS	ATP sulfurylase	AK099593	GGAAGGTGTCCTCAACCCAG	CATTGCACTTCAGTTGGCCC
CYS1	an isogene of cysteine synthase	AF073695	AGGAGAAGGGGCTGATCACT	CAGTCCAATGCCTGTGTTGC
CYS3	an isogene of cysteine synthase	AF073697	TTGTGGTCTTCCCGAGCTTC	CATGTTTTCGGCCTCCCTCT

F-box	F-box domain protein	AK072358	CCCAAGCCTTCTACATTACTA	GAAAGCATCTCCAGCCTCT
GTF	Putative glucosyltransferase	AK102212	TCTTAGAAGACTAGGCTTCAACATC	AGGAAATCTGCTGCCAAATAC
IPPI	Isopentenylpyrophosphate isomerase	AK065871	GAACTCCTACTCCAGCAAC	CAGCAGCACAAGAACAGAGT
LRR	Leucine-rich repeat family protein	AK122171	GCAATGCCGAATCTGTTG	GTAAGATCCAATTCCTCCAAGT
Lsi2	Silicon efflux transporter	AK101092	CGGGAGCCATCTGGGACTT	TTAATTGACTTGAGGTTGAGGAGC
MATE	Multi-drug and toxin extrusion transporter	LOC_Os05g48040.1	GCACCTGGGAGCCTATGTAA	TTGCCAGTTGCTGAAGAATG
NRAT1	Nramp aluminum transporter 1	AB525887	AAGATTCCAGTGTGGGCTGG	ATGAACTCCAGCTTCCGAGC
OsALS1	ABC transporter	AB625451	CGTATCAAGACCGGCAACCG	TCATGCCTCGCCAACTTGAT
PAPS	Purple acid phosphatase	AK070684	GCGAAGTGGAAGATTGCT	GGAGGGCTGGAGGTAGTT
PCS	phytochelatin synthase	AK065501	TCCAGCGCATCATAAGCCTC	CCCCCATTTCGTTTCCCTTG
SQS	Squalene synthase	AK063341	ACTATGTGGCAGGGCTAG	TAGGTGGTCATTACAGAGGA
ST1	Putative sulphate transporter 1	AF493792	GTACAAGGACCAGCCGATGT	ATGTCCTGGGGTATGCAGAG
ZFP	Zinc finger protein-like	AK071063	ACCGATGTCTCGCAGGAA	ATACCATCACGGGCAACC
